# Awareness of Vitamin D Deficiency among the General Population in Jeddah, Saudi Arabia

**DOI:** 10.1155/2019/4138187

**Published:** 2019-03-03

**Authors:** Lujain H. Alamoudi, Rinad Z. Almuteeri, Murooj E. Al-Otaibi, Dalia A. Alshaer, Samar K. Fatani, Maha M. Alghamdi, Osama Y. Safdar

**Affiliations:** ^1^Faculty of Medicine, King Abdulaziz University, Jeddah, Saudi Arabia; ^2^Pediatric Nephrology Center of Excellence, Faculty of Medicine, King Abdulaziz University, Jeddah, Saudi Arabia

## Abstract

**Background:**

During the last decade, vitamin D status was a major concern in the health and biomedical fields. This study assessed the level of awareness and knowledge regarding vitamin D and investigated the factors associated with the level of awareness such as knowing general facts about vitamin D, sources, benefit, and consequences of its deficiency.

**Methods:**

This cross-sectional study was conducted among the general population in Jeddah, Saudi Arabia, above the age of 18 years who were in 3 malls during the period of August and September 2017. A self-administrated questionnaire in paper forms was utilized and was divided into two parts to collect data. It was designed by the authors after revising the previous studies and validated by three pediatric consultants. An ethical approval was obtained from the ethical committee in King Abdulaziz University. We did an initial sample and tested it with the Cronbach test. The questionnaire had 2 parts with 19 questions. The first part was demographic information, and the second part was general information about vitamin D. An ethical approval was obtained from the ethical committee in King Abdulaziz University. Each participant received explanations about the aim of the study, and a verbal consent was taken from participants. The scoring system was as follows: for each right answer, 1 score was given and for wrong answer, 0 score was given, and the overall score was 14. The collected data were statistically analyzed using descriptive statistics using IBM SPSS statistics for windows version 20.0 (SPSS Inc., Chicago, IL).

**Result:**

Out of 1022 participants, 472 (46.1%) were aged 18–28 years, 830 (82.1%) were of Saudi ethnicity, 702 (68.7%) had a university degree, 275 (26.9%) attended high school, more than half were married (55.6%), and 54.4% had children. The majority of the participants agreed that vitamin D is important in the maintenance of bone and tooth health (88.4%). It is important in the maintenance of calcium and phosphates (76.6%), and it strengthens immunity (69.4%). Of the total participants, 86.2% were aware that sunlight exposure encourages vitamin D production in the skin. The overall knowledge mean score was 5.9 ± 1.2 (39.3%).

**Conclusion:**

This study highlighted a high level of inadequate knowledge of vitamin D deficiency among participants. There was a significant association between knowledge level and education level. The awareness of vitamin D deficiency is high regarding its benefits. The study revealed that participants who did not have children had the highest score for benefits. Nongovernment organizations and social workers may work together with government health-care organization to teach parents and children about the uses and benefits of vitamin D.

## 1. Introduction

During the last decade, there has been major concern about vitamin D status in the health and biomedical fields, and many studies have been conducted examining its benefits, use, and deficiency [1–6].

Internationally, vitamin D deficiency is a global health problem in both children and adults and is considered an epidemic [7, 8].

The Middle East and North African region which includes Saudi Arabia has a very high rate of vitamin D deficiency which reaches 81% among various age groups [1, 9]. Vitamin D has two biologically inert precursors: vitamin D3 and vitamin D2 [10].

Both precursors which come from sunlight exposure and diet are converted by two hydroxylations: one in the liver and the other in the kidney to form active 1, 25-dihydroxyvitamin D (calcitriol) [11, 12]. Metabolism of vitamin D also takes place in other tissues where the 1, 25(OH)2D produced serves a paracrine/autocrine function [13, 14]. If there is no proper dietary intake of vitamin D or proper exposure to sunlight, vitamin D deficiency will occur [10].

The role of vitamin D in calcium and phosphorus homeostasis and bone metabolism has been well recognized [8, 9]. The presence of vitamin D receptors in many cell types indicates that it has other physiologic functions. In children, it is associated with nutritional rickets, impaired growth, developmental delays, lethargy, and hypocalcemic seizures [15]. Lack of knowledge and awareness of vitamin D deficiency can be potential risk factors for the condition. Many studies show that there is lack of awareness and knowledge of vitamin D role and sources in several countries, including Hong Kong, Saudi Arabia, USA, and India [2, 13, 16–18].

This study aimed at assessing the level of awareness and knowledge of vitamin D and at investigating the factors associated with the level of awareness such as the knowledge in vitamin D sources, benefits, and consequences of its deficiency and overdose toxicity. It will also consolidate the results about public awareness and to check the gap of knowledge among the Saudi population.

## 2. Method

This cross-sectional study was conducted among general population who went to three malls during study duration, in Jeddah, Saudi Arabia. We used the software https://www.surveysystem.com/sscalc.htm.

To calculate the required sample size for Saudi population who is estimated to be 28 million with a confidence level of 95% and confidence interval of 5%, the calculated required sample size estimated to be 1022 self-administrated questionnaire was utilized in the paper form to collect the data on site. The inclusion criterion was age ≥18 years, and the exclusion criteria were age less than 18 and people working in the medical field including physician, nurses, technician, pharmacists, dentists, and medical students.

It was designed by the authors after revision of the previous studies and validated by three consultants: a general pediatrician, pediatric endocrinologist, and adult endocrinologist; an initial sample was done, and the reliability of the questionnaire items was tested by the Cronbach alpha test.

An ethical approval was obtained from the ethical committee in King Abdulaziz University. Each participant received explanations about the aim of the study, and a verbal consent was taken from participants.

The questionnaire consisted of two parts with twenty-four questions total. The first part collected demographic information (age, sex, nationality, educational level, and number of children), and the second part collected information regarding knowledge about vitamin D (benefits, deficiency, resources, and toxicity).

The score was given as follows: for each correct answer, 1 point was given and for an incorrect answer, 0 point was given, with the overall score being 14.

The collected data were statistically analyzed using descriptive statistics using IBM SPSS statistics for windows version 20.0 (SPSS Inc., Chicago, IL).

Parametric data were expressed as means, standard deviations, and minimums and maximums, while nonparametric data were expressed as numbers and percentages. We compared participants' knowledge of vitamin D among demographic groups using an independent *t*-test and a one-way analysis of variance test. A *p* value of less than 0.05 was considered statistically significant.

## 3. Result

The questionnaire was initially distributed randomly to 50 participants to assess reliability and validation. The Cronbach alpha value was 0.609 which is considered acceptable (Table 1). Out of 1022 total participants, 472 (46.1%) were aged 18–28 years, 830 (82.1%) were of Saudi ethnicity, 702 (68.7%) had a university degree, 275 (26.9%) completed high school, more than half of the participants were married (55.6%), and 54.4% had children (Table 2).

Awareness regarding the benefits of vitamin D is shown in Table 3. The majority of the participants agreed that vitamin D is important in the maintenance of bone and teeth (88.4%), it is important in the maintenance of calcium and phosphates (76.6%), and it strengthens immunity (69.4%) (Table 3).

Awareness of the sources of vitamin D is shown in Table 4. The majority of participants believe that sunlight exposure encourages vitamin D production in the skin (86.2%) and that people residing in cloudy areas are more prone to vitamin D deficiency (62.7%). Approximately 30.1% agreed that sun exposure never leads to vitamin D poisoning, and 14.4% agreed that people with dark skin are more prone to vitamin D deficiency (Table 4).

Awareness of vitamin D toxicity is shown in Table 5. Approximately 25.6% were aware that vitamin D toxicity can cause hypercalcemia (Table 5).

Table 6 shows the mean knowledge scores. The overall mean knowledge score was 5.9 ± 1.7 (39.3%). The mean knowledge score for benefits was 3.6 ± 1.2 (60%). The mean knowledge score for sources was 2.8 ± 1.6 (35%), and the mean knowledge score for toxicity was 0.3 ± 0.1 (30%) (Table 6).

In comparing age groups, there was no significant difference found among age groups for all knowledge domains (benefits, source, toxicity, and overall) (Table 7).

In comparing education levels, there was a significant difference found for overall knowledge score. Participants who attended high school had the highest score followed by those with a university degree (*p*=0.02). No significant difference was found among education levels for the other domains (benefits, sources, and toxicity) (Table 8).

When comparing marital status, the results revealed that no significant difference was found for sex and marital status among the other domains (benefits, sources, toxicity, and overall) (Table 9).

When comparing by whether or not a participant had children, the results revealed a significant difference in benefits, toxicity, and overall knowledge scores. Participants who did not have children had the highest score for benefits, deficiency, toxicity, and overall (*p*=0.06, *p*=0.014, and *p*=0.02, respectively). No significant difference was found regarding sources (Table 10).

Awareness regarding the benefits of vitamin D is shown in Table 3. The majority of the participants agreed that vitamin D is important in the maintenance of bone and teeth (88.4%) and calcium and phosphates (76.6%) and it strengthens immunity (69.4%) (Table 3).

Awareness of the sources of vitamin D is shown in Table 4. The majority of participants believe that sunlight exposure encourages vitamin D production in the skin (86.2%) and that people residing in cloudy areas are more prone to vitamin D deficiency (62.7%). Approximately 30.1% agreed that sun exposure never leads to vitamin D poisoning, and 14.4% agreed that people with dark skin are more prone to vitamin D deficiency (Table 4).

Awareness of vitamin D toxicity is shown in Table 5. Approximately 25.6% were aware that vitamin D toxicity can cause hypercalcemia (Table 5).

Table 6 shows the mean knowledge scores. The overall mean knowledge score was 5.9 ± 1.7 (39.3%). The mean knowledge score for benefits was 3.6 ± 1.2 (60%). The mean knowledge score for sources was 2.8 ± 1.6 (35%), and the mean knowledge score for toxicity was 0.3 ± 0.1 (30%) (Table 6).

In comparing age groups, there was no significant difference found among age groups for all knowledge domains (benefits, source, toxicity, and overall) (Table 7).

In comparing education levels, there was a significant difference found for overall knowledge score. Participants who attended high school had the highest score followed by those with a university degree (*p*=0.02). No significant difference was found among education levels for the other domains (benefits, sources, and toxicity) (Table 8).

When comparing marital status, the results revealed that no significant difference was found for sex and marital status among the other domains (benefits, sources, toxicity, and overall) (Table 9).

When comparing by whether or not a participant had children, the results revealed a significant difference in benefits, toxicity, and overall knowledge scores. Participants who did not have children had the highest score for benefits, deficiency, toxicity, and overall (*p*=0.06, *p*=0.014, and *p*=0.02, respectively). No significant difference was found regarding sources (Table 10).

## 4. Discussion

The results of the current study showed that almost two-thirds of the participants have an adequate level of knowledge about vitamin D benefits. Globally, there is there is a low level of awareness and knowledge regarding vitamin D which might be a possibly contributing factor to high levels of vitamin D deficiency worldwide. In a study conducted in United Kingdom, more than two-thirds of participants had a low level of knowledge about the commonest symptoms of vitamin D deficiency [2].

Another study in India study showed that more than half of participants had a low level of knowledge about vitamin D deficiency in antenatal mothers [19] and another study reported in Saudi Arabia revealed that 40% of the mothers attending primary health centers in Al-Ahsa had a low level of knowledge about vitamin D supplementation to their infants [20].

Exposure to sunlight is an important source of vitamin D. According to a study, vitamin D produced in the skin may last longer in the blood than with vitamin D from diet [21]. There are a number of factors that decrease the skin's production of vitamin D like aging, dark skin, and sunscreen application [22–24].

Dietary sources that naturally contain vitamin D are limited, including egg yolk, fish, and liver. In some countries, a number of foods are fortified with vitamin D, like milk, and cereals [25].

The results of this study revealed that participants need an improvement of knowledge about the sources of vitamin D, and only a third of the participants had accurate information regarding vitamin D resources.

Similarly, Al-Agha's study showed a good level of knowledge about sun exposure and dietary intake as vitamin D sources, but many people had poor knowledge about the safe times for sun exposure and many did not have time due to their working lifestyle [26].

Similar results were found in a French study where 72% of participants reported sun exposure as the main source of vitamin D and 50–60% of participants reported the right food sources like fatty fish [27].

The current study revealed that participants had a good level of knowledge regarding the benefits of vitamin D, where more than two-thirds were knowledgeable that vitamin D helps with bone health, tooth health, immune health, and muscle health. These results were higher than what was found in an Australian study (43.0%) [28] and lower than the findings in a French study (78.1%) [27].

Comparison of knowledge scores among sociodemographic groups revealed significant differences in knowledge scores for education level and for having children. Those who had a university degree and those who do not have children showed a higher level of knowledge than others. A similar result was found in Poland where the better educated mothers had a greater knowledge [29].

On the other hand, there was no significant difference in knowledge scores regarding age, sex, and marital status. In an Australian study, the authors reported that being female, older, and having a higher educational degree were indicators for having a higher level of knowledge [28]. Similar findings were reported in a French study that included a higher monthly income as a predictor [27]. While in Al-Agha's study, the low level of knowledge was not associated with education level [26], and in a United Kingdom study, older people showed a low level of knowledge [2].

## 5. Strengths and Limitations

Our study assessed multiple health aspects of vitamin D regarding its benefits, resources, and toxicity in Saudi Arabia with a relatively large sample size. As a limitation, many other studies were conducted regarding the awareness of vitamin D; however, we believe there is still a need for further awareness studies regarding vitamin D due to the gap of knowledge witnessed.

## 6. Conclusion

The study highlighted the high level of inadequate knowledge regarding vitamin D among study participants. There is a significant association between level of knowledge and education level. More campaigns need to be held to educate people sufficiently regarding the dietary sources and established health benefits of vitamin D for bones, muscles, and immune system. Nongovernment organizations and social workers may work together with government health-care organizations to teach parents and children about the uses and benefits of vitamin D. Further, there is a need to conduct interventions and qualitative studies to determine the level of awareness, to determine the underlying reasons for this poor level, and to fill the knowledge gaps. All of these measures will help to improve vitamin D status and the health problems related to its deficiency or toxicity among the Saudi population.

## Figures and Tables

**Table 1:** Cronbach test.

Total factor				
*Reliability statistics*				
Cronbach's alpha	*N* of Items			
0.608	14			
*Item-total statistics*				
	Scale mean if item deleted	Scale variance if item deleted	Corrected item-total correlation	Cronbach's alpha if item deleted
v1, vitamin D is internally made by the sun	43.45	20.286	0.051	0.617
v2, vitamin D is used to treat rickets	43.70	19.120	0.187	0.599
v3, vitamin D is important in maintaining calcium and phosphate	43.51	17.907	0.330	0.573
v4, vitamin D is important in maintaining bone and teeth	43.36	18.214	0.375	0.569
v5, vitamin D helps strengthen immunity	43.78	17.226	0.442	0.552
v6, vitamin D is higher in animal meat than vegetables and fruits	44.17	17.816	0.306	0.577
v9, vitamin D helps strengthen muscles	44.15	17.222	0.357	0.566
v11, residents of cloudy areas(full of cloud) are more likely to lake vitamin D(because there is not enough sunlight)	44.44	19.649	0.049	0.631
v12, darker skin is more prone to vitamin D deficiency	44.85	18.967	0.229	0.592
v13, vegetarians do not consume enough vitamin D and are more prone to deficiency	44.32	18.800	0.251	0.589
v15, fat-free diet may be a cause of vitamin D deficiency	44.47	18.106	0.327	0.575
v16, frequent exposure to the sun does not lead to the poisoning of vitamin D	44.88	19.286	0.145	0.607
v17, the use of sunscreen cream may be cause of vitamin D deficiency	44.95	19.179	0.196	0.598
v19, vitamin D poisoning may lead to hypercalcemia leading to calcinosis	44.46	19.723	0.178	0.600

**Table 2:** Participant characteristics.

Variables	*N*	%
*Age*		
18–28	472	46.1
29–39	223	21.8
40–50	220	21.5
51–60	97	9.5
More than 60	10	1.0
*Nationality*		
Saudi	830	81.2
Non-Saudi (Egyptian, Iraqi, Sudanese, and Yemen)	192	18.8
*Education*		
University	702	68.7
High school	275	26.9
Intermediate	36	3.5
Elementary	9	0.9
*Marital status*		
Married	568	55.6
Single	410	40.1
Divorced	32	3.1
Widowed	12	1.2
*Have children*		
Yes	556	54.4
No	466	45.6

**Table 3:** Awareness regarding the benefits of vitamin D.

Variables	*N*	%
Vitamin D is used to treat bone disease and rickets		
No	318	31.1
Yes	704	68.9
Vitamin D is important in the maintenance of calcium and phosphates		
No	239	23.4
Yes	783	76.6
Vitamin D is important in the maintenance of bone and teeth		
No	119	11.6
Yes	903	88.4
Vitamin D helps to strengthen immunity		
No	313	30.6
Yes	709	69.4
Vitamin D helps to strengthen muscles		
No	397	38.7
Yes	625	61.2

**Table 4:** Awareness regarding sources of vitamin D.

Variables	*N*	%
Sun exposure encourages vitamin D production in the skin		
No	140	13.7
Yes	882	86.3
Vitamin D is found in animal meat but not in vegetables and fruits		
No	806	78.9
Yes	216	21.1
People residing in cloudy areas are more prone to vitamin D deficiency		
No	381	37.3
Yes	641	62.7
Frequent sun exposure does not lead to vitamin D poisoning		
No	714	69.9
Yes	308	30.1
Use of sunscreen creams may be a cause of vitamin D deficiency		
No	843	82.5
Yes	179	17.5
A fat-free diet may be a cause of vitamin D deficiency		
No	813	79.5
Yes	209	20.5
Dark skin is more prone to vitamin D deficiency than fairer skin		
No	875	85.6
Yes	147	14.4
Vegetarians are more likely to have vitamin D deficiency than nonvegetarians		
No	780	76.3
Yes	242	23.7

**Table 5:** Awareness regarding consequences of vitamin D toxicity.

Variables	*N*	%
Hypercalcemia in the blood issues		
No	760	74.4
Yes	262	25.6

**Table 6:** Vitamin D knowledge scores.

Variables	Mean ± SD	Range (min-max)	Total	%
Benefits	3.6 ± 1.2	(0–6)	6	60.0
Sources	2.8 ± 1.6	(0–8)	8	35.0
Toxicity	0.3 ± 0.1	(0-1)	1	30.0
Overall	5.9 ± 1.7	(0–16)	5	39.3

**Table 7:** Knowledge score by age.

Variables	Mean ± SD	*p* value
Benefits		
18–28	2.71 ± 1.63	0.09
29–39	2.74 ± 1.56	
40–50	2.83 ± 1.60	
51–60	2.87 ± 1.26	
More than 60	3.30 ± 1.95	
Sources		
18–28	3.58 ± 1.27	0.62
29–39	3.86 ± 1.25	
40–50	3.60 ± 1.32	
51–60	3.59 ± 1.28	
More than 60	3.70 ± 1.25	
Toxicity		
18–28	0.45 ± 0.29	0.12
29–39	0.39 ± 0.19	
40–50	0.43 ± 0.25	
51–60	0.43 ± 0.24	
More than 60	0.42 ± 0.20	
Overall		
18–28	6.81 ± 2.69	0.63
29–39	7.14 ± 2.62	
40–50	6.99 ± 2.71	
51–60	6.92 ± 2.32	
More than 60	7.30 ± 3.02	

*p* values <0.05 are considered statistically significant.

**Table 8:** Knowledge score by education level.

Variables	Mean ± SD	*p* value
Benefits		
University	2.83 ± 1.58	0.18
High school	2.57 ± 1.55	
Intermediate	3.00 ± 1.82	
Elementary	2.44 ± 1.13	
Resources		
University	3.69 ± 1.25	0.08
High school	3.57 ± 1.33	
Intermediate	3.61 ± 1.39	
Elementary	2.89 ± 1.45	
Overdose		
University	0.45 ± 0.28	0.05
High school	0.41 ± 0.21	
Intermediate	0.44 ± 0.25	
Overall		
University	7.09 ± 2.65	0.02^*∗*^
High school	6.55 ± 2.58	
Intermediate	7.14 ± 2.87	
Elementary	5.67 ± 1.74	

^*∗*^ *p*-values <0.05 are considered statistically significant.

**Table 9:** Knowledge score by marital status.

Variables	Mean ± SD	*p* value
Benefits		
Married	2.77 ± 1.54	0.06
Single	2.76 ± 1.65	
Divorce	2.72 ± 1.53	
Widow	2.97 ± 0.99	
Resources		
Married	3.73 ± 1.26	0.98
Single	3.54 ± 1.29	
Divorce	3.34 ± 1.38	
Widow	3.83 ± 1.11	
Overdose		
Married	0.42 ± 0.22	0.28
Single	0.46 ± 0.29	
Divorce	0.44 ± 0.25	
Widow	0.51 ± 0.58	
Overall		
Married	7.02 ± 2.62	0.41
Single	6.82 ± 2.73	
Divorce	6.56 ± 2.45	
Widow	7.67 ± 2.02	

*p* values <0.05 are considered statistically significant.

**Table 10:** Knowledge score by parental status.

Variables	Mean ± SD	*p* value
Benefits		
Yes	2.77 ± 1.52	0.002^*∗*^
No	2.90 ± 1.89	
Not married	2.71 ± 1.57	
Resources		
Yes	3.71 ± 1.25	0.50
No	3.83 ± 1.28	
Not married	3.48 ± 1.30	
Overdose		
Yes	0.415 ± 0.22	0.001^*∗*^
No	0.47 ± 0.34	
Not married	0.45 ± 0.28	
Overall		
Yes	6.99 ± 2.58	0.02^*∗*^
No	7.46 ± 3.02	
Not married	6.67 ± 2.61	

^*∗*^ *p*-values<0.05 are considered statistically significant.

## Data Availability

The data used to support the findings of this study are available from the corresponding author upon request.
